# Cardiopulmonary Exercise Testing after Surgical Repair of Tetralogy of Fallot—Does Modality Matter?

**DOI:** 10.3390/jcm13051192

**Published:** 2024-02-20

**Authors:** Benedetta Leonardi, Fabrizio Sollazzo, Federica Gentili, Massimiliano Bianco, Elettra Pomiato, Stefani Silva Kikina, Rachel Maya Wald, Vincenzo Palmieri, Aurelio Secinaro, Giulio Calcagni, Gianfranco Butera, Ugo Giordano, Giulia Cafiero, Fabrizio Drago

**Affiliations:** 1 Department of Pediatric Cardiology, Cardiac Surgery and Heart Lung Transplantation, Bambino Gesù Children’s Hospital, IRCCS, 00165 Rome, Italy; elettra.pomiato@gmail.com (E.P.); giulio.calcagni@opbg.net (G.C.); gianfranco.butera@opbg.net (G.B.); fabrizio.drago@opbg.net (F.D.); 2 Unità Operativa Complessa di Medicina dello Sport e Rieducazione Funzionale, Fondazione Policlinico Universitario Agostino Gemelli IRCCS, Università Cattolica del Sacro Cuore, 00168 Rome, Italy; fabrizio.sollazzo@policlinicogemelli.it (F.S.); massimiliano.bianco@policlinicogemelli.it (M.B.); vincenzo.palmieri@policlinicogemelli.it (V.P.); 3 Unit of Sport Medicine, Bambino Gesù Children’s Hospital, IRCCS, 00165 Rome, Italy; federica.gentili@opbg.net (F.G.); ugo.giordano@opbg.net (U.G.); giulia.cafiero@opbg.net (G.C.); 4 Department of General Surgery, Southend University Hospital, Mid and South Essex NHS Foundation Trust, Westcliff-on-Sea SS0 0RY, UK; stefani.kikina@nhs.net; 5 Toronto General Hospital Research Institute (TGHRI), Toronto, ON M5G 2N2, Canada; rachel.wald@uhn.ca; 6 Advanced Cardiothoracic Imaging Unit, Department of Imaging, Bambino Gesù Children’s Hospital, IRCCS, 00168 Rome, Italy; aurelio.secinaro@opbg.net

**Keywords:** cardiopulmonary exercise testing, congenital heart disease, cycle ergometer, treadmill, protocol selection

## Abstract

Background: Despite a successful repair of tetralogy of Fallot (rToF) in childhood, residual lesions are common and can contribute to impaired exercise capacity. Although both cycle ergometer and treadmill protocols are often used interchangeably these approaches have not been directly compared. In this study we examined cardiopulmonary exercise test (CPET) measurements in rToF. Methods: Inclusion criteria were clinically stable rToF patients able to perform a cardiac magnetic resonance imaging (CMR) and two CPET studies, one on the treadmill (incremental Bruce protocol) and one on the cycle ergometer (ramped protocol), within 12 months. Demographic, surgical and clinical data; functional class; QRS duration; CMR measures; CPET data and international physical activity questionnaire (IPAQ) scores of patients were collected. Results: Fifty-seven patients were enrolled (53% male, 20.5 ± 7.8 years at CPET). CMR measurements included a right ventricle (RV) end-diastolic volume index of 119 ± 22 mL/m^2^, a RV ejection fraction (EF) of 55 ± 6% and a left ventricular (LV) EF of 56 ± 5%. Peak oxygen consumption (VO_2_)/Kg (25.5 ± 5.5 vs. 31.7 ± 6.9; *p* < 0.0001), VO_2_ at anaerobic threshold (AT) (15.3 ± 3.9 vs. 22.0 ± 4.5; *p* < 0.0001), peak O_2_ pulse (10.6 ± 3.0 vs. 12.1± 3.4; *p* = 0.0061) and oxygen uptake efficiency slope (OUES) (1932.2 ± 623.6 vs. 2292.0 ± 639.4; *p* < 0.001) were significantly lower on the cycle ergometer compared with the treadmill, differently from ventilatory efficiency (VE/VCO_2_) max which was significantly higher on the cycle ergometer (32.2 ± 4.5 vs. 30.4 ± 5.4; *p* < 0.001). Only the VE/VCO_2_ slope at the respiratory compensation point (RCP) was similar between the two methodologies (*p* = 0.150). Conclusions: The majority of CPET measurements differed according to the modality of testing, with the exception being the VE/VCO_2_ slope at RCP. Our data suggest that CPET parameters should be interpreted according to test type; however, these findings should be validated in larger populations and in a variety of institutions.

## 1. Introduction

Patients with congenital heart disease (CHD) often have an altered perception of their limitations as a result of long-term adaptation to their diminished physical capacity [1,2,3]. Several studies have shown a poor correlation between the subjective evaluation of exercise intolerance symptoms and exercise capacity assessed objectively with cardiopulmonary exercise testing (CPET) [4,5,6,7].

Tetralogy of Fallot is the most common form of cyanotic congenital heart disease at birth and occurs at a rate of 0.28–0.48 per 1000 live births [8]. Despite the relatively good clinical outcomes of patients with repaired tetralogy of Fallot (rToF), these patients commonly experience exercise limitations which typically worsen over time [5]. Exercise intolerance in this population is multifactorial and has been shown to relate to pulmonary regurgitation (PR), impaired lung function, chronotropic impairment and/or ventricular dysfunction [6].

Many studies have explored the importance of CPET parameters in rToF, both maximal (peak oxygen consumption) and sub-maximal (oxygen uptake efficiency slope and ventilatory efficiency) [4,7,9,10]. Aerobic capacity (peak VO_2_) and ventilatory equivalent for carbon dioxide (VE/CO_2_) slope have been shown to be useful predictors of early mortality after pulmonary valve replacement (PVR) and have been associated with hospital admission and death in a long-term follow-up of rToF patients [11,12]. Despite the clinical value of this test there is still no consensus regarding the type of equipment (cycle ergometer vs. treadmill) or protocol which is best suited to investigation of patients with rToF. Of note, multiple factors can impact CPET results, including factors such as patient familiarity with equipment or protocol, age, BMI, sex, fitness level and coexisting lung pathology. That is to say, if the same individual completed a CPET on the treadmill and on the cycle ergometer, it is likely that different values of peak VO_2_ would be recorded. Likewise, the CPET performed on the same equipment but using different protocols may result in different values of peak VO_2_. The treadmill test induces greater stimulation of the heart and the lung compared with cycle ergometers. It is also a more natural form of exercise, is more suitable for children and produces a higher peak VO_2_ [13,14]. In fact, in the exercise tests using a cycle ergometer, untrained subjects usually request to terminate the test due to quadriceps femoris muscle fatigue, thereby achieving 5%–20% lower peak VO_2_ on average compared with that obtained using treadmill exercise among both healthy subjects and patients with heart disease [14,15,16,17]. The influence of stroke volume on cardiac output exerts a significant impact on peak VO_2_ during cycling and running and stroke volume under the stimulation of treadmill exercise is larger than that with cycle ergometers. The heart rate (HR) peak of subjects performing treadmill exercise is 10–25 times higher than that of those performing the cycle ergometer exercise. The peak systolic blood pressure (SBP) × HR is also higher in the treadmill group, indicating higher myocardial oxygen consumption.

On the other hand, a scientific statement issued by the American Heart Association in 2009 estimated the incidence of fatal adverse events and events requiring medical intervention during exercise testing to be <0.01 using a cycle ergometer and <0.2 using treadmill [17]. In addition, step protocols, especially with large and unequal work increments, have been associated with less accurate estimate of exercise capacity [18], a weaker relationship between exercise test time and ischemia, and a narrower distribution of time before the onset of ST-segment depression [13,18,19,20,21]. Instead, a cycle ergometer allows for precise quantification of external work rate and easier evaluation of useful parameters during effort (e.g., blood samples, blood pressure), and reduces electrocardiographic artifacts and the risk of falling [18].

Moreover, the cycle ergometer could make it easier for clinicians to identify relevant parameters such as ventilatory anaerobic threshold (VAT) [20]. For all these reasons, ramp protocols, which involve constant increments in work rate at intervals of less than 60 s, are generally preferred for the CPET and have been shown to be feasible even in children with respiratory disease [22]. However, the current guidelines for the CPET do not provide any specific recommendations for cardiac pathologies, either ischemic or congenital, or which exercise modality to use for better results. Therefore, exercise selection relies mainly on local conditions and patient cooperation during the exercise. Furthermore, a few studies provide references for CPET values for both treadmill and cycle ergometry tests in normal individuals and patients with coronary heart disease and heart failure [14,16,17], but not in patients with CHD. Both methods can be safely used in rToF patients [4,7,9,10], but there have been no studies on the differences in values in the CPET parameters between the two methods or which method is best to evaluate oxygen consumption and to stratify the risk in relation to the degree of pathology of the patient. Therefore, in this study we aimed to compare cardiopulmonary results between the two methods (the Bruce incremental protocol on a treadmill and the ramped protocol on a cycle ergometer) in asymptomatic patients with rToF in order to explore whether submaximal and maximal cardiorespiratory parameters are influenced by test modality.

## 2. Materials and Methods

Study inclusion criteria were rToF patients with follow-up at our institution, age > 12 years and asymptomatic status. Exclusion criteria were NYHA functional class > I; involvement in high-level physical activities, classified according to the international physical activity questionnaire (IPAQ); and changes in clinical status and/or in the patient’s weight (≥5 kg) in the period between the two CPETs. In addition, we excluded pregnant patients as well as those with previous pulmonary valve replacement (PVR). The research protocol included a complete clinical evaluation, a 12-lead electrocardiogram (to exclude the possibility of an arrhythmia), an echocardiogram and a 24 h Holter monitoring and cardiac MRI. These tests were used to exclude the most relevant contraindications to the CPET and to demonstrate any relevant changes in clinical status since the previous clinical examination (within the last twelve months). Patients with a BMI > 30 were considered obese. The study was approved by the Ethics Committee of the Bambino Gesù Children’s Hospital, IRCCS (Prot. Number 341/2015), and all subjects signed an informed consent form. The study was conducted in accordance with the Declaration of Helsinki.

### 2.1. Cardiopulmonary Exercise Testing

All patients with rToF, in a total cohort of 330 subjects, who were able to perform the CPET on the treadmill and agreed to repeat the above-mentioned test with the cycle ergometer within the same year, were enrolled in the years 2021–2022. The participants were instructed to refrain from strenuous physical activity on testing days and not to eat for at least 2 h prior to the test. CPETs were performed by a senior sports medicine doctor (FG) or a cardiologist (EP), both experienced in CHD. The standard incremental Bruce protocol was applied during the treadmill CPET, whereas the cycle ergometer CPET (Cosmed Quark PFT Cycle ergometer Technogym bike 1000 Med) was performed using a ramp protocol, with individualized workload increments calculated by the Wassermann equation [18], to be completed in a time range of 8 to 12 min. Patients were asked to keep up a constant pace of 65–70 revolutions per minute (rpm). Breath-by-breath expired gas was recorded and analyzed using a calibrated metabolic measurement system. Spirometry was performed prior to each CPET. Blood pressure and pulse oximetry were recorded every 2–3 min and at peak exercise. All patients were strongly verbally encouraged throughout the test to maintain the cadence of ±5 rpm and to achieve maximal effort. In both tests, patients exercised until volitional fatigue or until the occurrence of symptoms and/or appearance of threatening arrhythmias (supraventricular or ventricular tachycardia, atrial fibrillation). Tests were considered maximal when at least two of the following criteria were achieved: (1) failure to maintain the work rate, (2) respiratory exchange ratio (RER) > 1.1), (3) maximal HR > 85% of age-predicted maximum (220-age) and (4) occurrence of a VO_2_ plateau (VO_2_ increase ≤150 mL/min over the last 30s of the test). Peak VO_2_ was calculated in both tests as the 15 s average of the highest VO_2_ achieved during the test.

For the CPET data, percentages of the predicted values of peak VO_2_ were determined using Burstein et al. reference values for ramp cycle ergometer tests in patients younger than 18 years [23]. Wasserman equations were used for both tests in those older than 18 years [18]. The percentage of 80% of peak VO_2_ was considered the threshold of normality. The following maximal cardiopulmonary parameters were collected and analyzed: peak oxygen uptake (peak VO_2_), peak oxygen uptake normalized for body weight (peak VO_2_/kg) and the relationship between minute ventilation and carbon dioxide production (VE/VCO_2_ slopes) measured at respiratory compensation point (RCP). In addition, the following submaximal cardiopulmonary parameters were analyzed: oxygen pulse (i.e., oxygen uptake to heart rate ratio), oxygen uptake efficiency slope (OUES), anaerobic threshold (AT) and ventilatory efficiency (VE/VCO_2_) at AT. Patient weight and height were recorded. Body surface area (BSA) and body mass index (BMI) were calculated. The formula of DuBois and DuBois was used to calculated BSA.

### 2.2. Statistical Analysis

Descriptive statistics are expressed as mean with standard deviations or median with interquartile range (IQR) for continuous variables and as counts and percentages for categorical variables. Normality was assessed using the Shapiro–Wilk test. For the comparison of normally distributed continuous variables, the independent samples t-test was used, and in the case of skewed distribution, the Mann–Whitney U-test or the Krusal–Wallis test was applied, as appropriate. All statistical analyses were performed using IBM SPSS Statistics 20.

## 3. Results

### 3.1. Baseline Characteristics

A total of 57 rToF patients (mean age 20.5 ± 7.8 years at CPET; age range 12.7–40.7 years) who underwent corrective surgery during childhood were included in the study. The most frequent type of repair in our population was the transannular patch (*n* = 48, 84%). The mean age at repair was 10.7 ± 10.7 (median 6.6; IQR 0.8–49.4) months. The echocardiographic evaluation revealed elevated right ventricular (RV) pressure in the presence of pulmonary arteries/right ventricle outflow tract stenosis in four patients (50 mmHg) and normal RV pressure in the remaining. No patients had aortic and/or mitral stenosis/insufficiency and only 4 patients had moderate tricuspid regurgitation (the other 19 had mild tricuspid regurgitation while the remaining 34 did not have tricuspid regurgitation). Only two patients were obese (≥30 kg/m2). Demographic and imaging data are shown (Table 1). No episodes of ventricular arrhythmias occurred during the CPET.

### 3.2. Comparison between the Treadmill Bruce Protocol and the Ramp Cycle Ergometer Protocol

**Figure 1:**
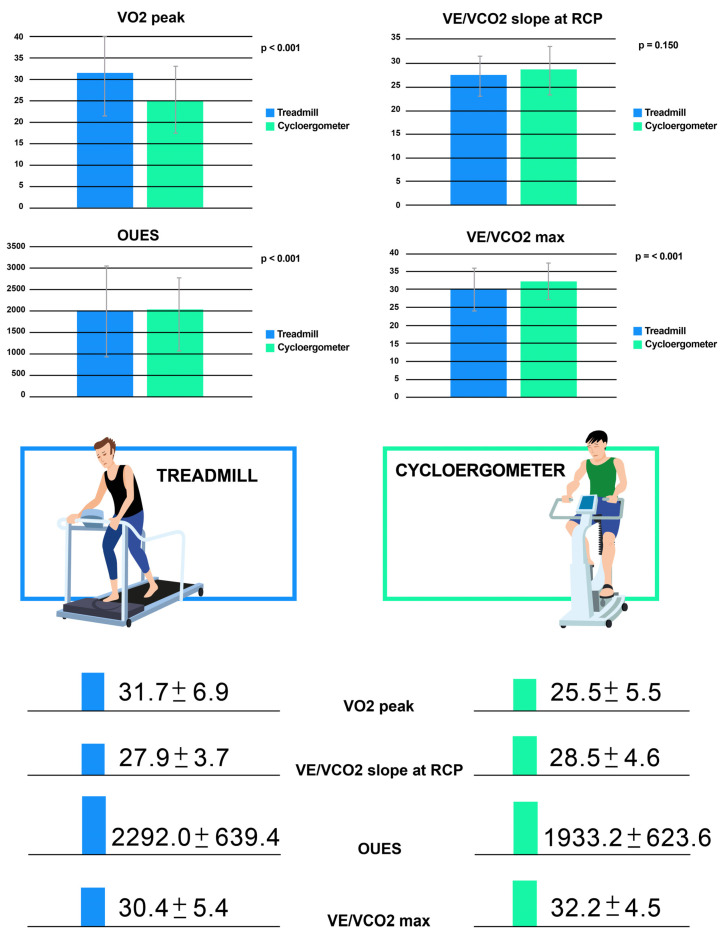
The differences of the following parameters between the cycle ergometer and treadmill: VO_2_ peak, VE/VCO_2_ max, VE/VCO_2_ slope at RCP and OUES. Legend: OUES: oxygen uptake efficiency slope; RCP: respiratory compensation point; VE/VCO_2_: ventilatory equivalent for CO_2_; VE: ventilation; VO_2_: oxygen consumption.

Comparison of values between the two test modalities is shown in detail (Table 2). The duration of each modality was significantly different, with longer duration seen on the treadmill, probably due to a greater familiarity with this type of exercise. Almost all maximal and submaximal indexes of performance (peak VO_2_, peak VO_2_/kg, age-predicted peak VO_2_, VO_2_ at AT, OUES, oxygen pulse) were significantly lower in the ramp cycle ergometer tests than in the Bruce treadmill tests (*p* < 0.05 for all) (Table 2; Figure 1). VO_2_ max was nearly 20% lower in the cycle ergometer test (*p* < 0.001; Table 2), while the predicted VO_2_ values obtained using the Wasserman equation in the adult patient were 21% lower in the cycle ergometer test. Predicted VO_2_ values obtained using Burstein’s equation in pediatric patients were 17% lower in the cycle ergometer test compared with the treadmill (both with a *p* < 0.001; Table 2). In contrast, no significant differences were observed between the two test modalities with regards to the VE/VCO_2_ slope at RCP (*p* = 0.150; Table 2). Spirometry values, measured prior to exercise, were not consistent with abnormal lung function and values did not differ significantly between the two methods.

## 4. Discussion

To the best of our knowledge, this is the first article on rToF patients that evaluates the possible intra-patient differences in CPET parameters between the cycle ergometer and treadmill in a population with rToF. We have documented that both methods are feasible and safe to assess the functional capacity of rToF patients. In particular, no differences in the incidence of arrhythmia events were present in either modality, although it is well known that these patients are at higher risk of arrhythmias [24]. In addition, the fundamental novelty of our results is the fact that almost all maximal and submaximal CPET parameters, except the VE/VCO_2_ slope at RCP, were dependent on the mode of exercise in the rToF patients. This is of considerable importance, for two reasons. Firstly, the interpretation of the CPET also requires an understanding of all the other parameters in addition to the VO_2_ peak [7,9,12,25]. Therefore, we can say that we now understand how these parameters vary in relation to the method of exercise we use in patients with CHD, such as ToF, in which the CPET is used for both arrhythmic risk stratification and to assess the indication for PVR [26,27,28]. Secondly, the similar values of the VE/VCO_2_ slope at RCP between the two modalities proves that the treadmill with Bruce protocol can provide an accurate estimate of the VE/VCO_2_ slope even if it derives from less constant, larger and unequal increments in work rate compared with the cycle ergometer [29]. This fact has only been documented in a few studies of adult heart failure patients with reduced ejection fraction [14,16]. To date, no data exist for a population with either CHD or ToF, in which the VE/VCO_2_ slope had been shown to be a prognostic factor [9,12,30]. Therefore, a proper interpretation of CPET results for risk stratification in ToF would be optimized by more precise knowledge of the impact of the mode of exercise on these prognostic markers [26].

### 4.1. Peak Oxygen Consumption

We have shown that the compensated patients with rToF (NYHA class I) without significant depression of ventricular function achieved a higher peak VO_2_ on the treadmill compared with the bicycle, in agreement with previous studies of healthy subjects [31,32,33] and adults with ischemic heart disease and heart failure [14,16]. In our study, VO_2_ max was nearly 20% lower in the cycle ergometer test compared with the treadmill, falling within the percentage documented by Mazaheri et al. for heart failure patients with severely reduced ejection fraction [14]. As documented in a small number of studies in the literature, the VO_2_ max reduction in healthy and ischemic heart disease patients varied greatly from 5 to 23%, probably due to inter-study and inter-individual variabilities [1,13,16,34,35,36]. This is the first evidence of how the average percentage of oxygen consumption varies in a population with compensated ToF. In addition, given that age and gender affect oxygen consumption, we also evaluated the predicted VO_2_ value in both pediatric and adult patients. Interestingly, it was 21% lower in the cycle ergometer test compared with the treadmill test in adult patients using the Wasserman equation, differently from the pediatric population in which the difference was smaller using Burstein’s equation, standing at around 17%. This result could be due to the fact that adolescents are more active than adults and perceive quadriceps femoris muscle fatigue later, making it a better test; such data should be taken in consideration when deciding the best method to perform the CPET in a rToF patient. Finally, given that the importance of correct assessment of peak VO_2_ and percentage of predicted peak VO_2_ as prognostic factors and for decision making is well known, and that peak VO_2_ is a crucial parameter in heart failure survival scores, it could be useful to have reference predicted peak VO_2_ values for both exercise test modes, and for each type of CHD.

### 4.2. Ventilatory Efficiency

The VE/VCO_2_ slope at RCP, which, in previous studies, has been identified as a powerful predictor of reduced cardiac output during exercise, cardiac-related hospitalization and death [11,12] was similar in the two methods in our study. Traditionally, the VE/VCO_2_ slope is more reliable when measured on the cycle ergometer during a ramp protocol [29]. However, in agreement with studies performed on adults with heart failure [14,16,37], our study documented that with the treadmill we can also achieve a “realistic” VE/VCO_2_ slope at RCP value. These data could be of considerable importance, given that one of the main limitations of the treadmill CPET is based on the belief that the VE/VCO_2_ slope at RCP value could differ between test modalities. Therefore, a multicenter study involving a larger number of patients should be performed to confirm these data. This could change current beliefs and, consequently, existing clinical practice.

### 4.3. Exercise Test Intensity Measures

In our cohort, the peak RER, which is the most valuable indicator of an individual’s effort in the CPET, was not significantly different between the two modalities, despite the different VO_2_ max values. Our results disagree with previous studies on the comparison between treadmill and cycle ergometer tests, performed in an adult population with heart failure [14,16,37]. This could be due to the age and severity of the pathology of the population considered. It is possible that young patients with ToF but without significant depression of the ventricular function obtain different CPET values on the two exercise test methods despite exerting similar levels of effort. Our results also demonstrate that achieving a maximal CPET is also possible with a cycle ergometer, by tailoring the ramp protocol to the single patient. In this setting, the relationship between O_2_ consumption and CO_2_ production was maintained despite the fact that absolute volumes of O_2_ and CO_2_ were reduced when exercising on the cycle ergometer compared with the treadmill.

### 4.4. Peak O_2_ Pulse and OUES

Our study also included peak O_2_ pulse and OUES, because we believed that, even though they are not commonly used in clinical practice, they could potentially help to outline the cardio-functional situation of ToF patients. Tsai et al. demonstrated that an indexed OUES/BSA value of <1.03 predicts hospitalization in children with rToF [25]. Peak O_2_ pulse, represents the change in stroke volume during exercise, given that it is the quotient of VO_2_ and heart rate. Therefore, an early plateau of peak O_2_ pulse could discriminate which rToF patients have limitations regarding the capacity of the cardiovascular system to increase stroke volume. Thus, it can identify which patients are at risk of developing RV decompensation. In fact, it is well known that a decrease in cardiac reserve (which documents the chronotropic insufficiency) is associated with poor prognosis in CHD [2]. In addition, the achievement of maximal effort is not required, as this approach may be an important alternative to peak VO_2_ for patients with a severe heart disease or those who are very young and are unable to exercise to a peak level. Finally, these values incorporate the evaluation of lung factors such as dead space and ventilation capabilities, given that they measure the amount of oxygen extracted relative to the amount of ventilation. In fact, we tend to focus mostly on peak VO_2_, not taking into consideration that not all patients reach a maximal effort and that, in this situation, the submaximal parameters of the CPET available are equally important in the prognosis of these patients.

### 4.5. Overall Summary

CPET measurements may vary considerably according to many factors (body size, gender, level of ordinary activities, familiarity with an activity and preconditioning with one activity over another, age and type of protocol used). We believe that our research provides an idea of how much individual parameters can vary from one type of equipment to another and this allows clinicians to decide, at each medical examination, which method is more appropriate for the patient’s clinical conditions. This is of great importance since the correct determination of peak VO_2_ represents a very relevant issue in patients with CHD (and specifically in rToF) due to the fact that peak VO_2_ is the key parameter for estimating exercise capacity as an expression of myocardial reserve. A reduction in objective exercise capacity is one of the criteria for referring rToF patients for PVR in asymptomatic individuals with severe PR, RV dilation and/or dysfunction [11,27,28]. In addition, peak VO_2_ is a well-known powerful predictor of cardiac-related mortality and hospitalization in rToF patients [2,12,26].

In a different clinical context, CPET results are considered one of the most valuable parameters for sports participation eligibility in rToF patients. For example, the Italian guidelines for pre-participation screening consider objective exercise tolerance, as measured by the CPET, as a criterion for competitive sports clearance, with a minimum peak VO_2_ value of 80% of the predicted, and a normal VE/VCO_2_ slope value [38].

The data that emerged from our study, which could be fundamental to radically changing the management of the follow-up of rToF patients’, need to be confirmed in a wider population in order to clarify the extent to which the degree of dilatation and/or dysfunction, as well as the level of physical activity, impacts the difference in CPET parameters in the two methods. Finally, given that the decline in VO_2_ max over time is more pronounced in children with CHD compared with healthy matched controls [39], it would be interesting to know if this decrease over time is similar in the two methods.

### 4.6. Limitations

The rToF population examined was a selected asymptomatic population without significant depression of RV function. Therefore, by focusing on patients in the “best possible clinical state”, this study was able to give an overview of how much the method of performing CPET impacts this subclass of patients; thus, it fails to represent the overall rToF population. We did not include the ramp treadmill and the step cycle ergometer protocols in the comparison, because we do not use them frequently. Thus, this study focused only on two of the methods of performing CPET.

## 5. Conclusions

Both the cycle ergometer and treadmill are feasible methods of performing CPET in young asymptomatic rToF patients. Almost all CPET values obtained were significantly different between the two methods, except for the VE/VCO_2_ slope at RCP. These data must be taken into consideration in choosing the ideal method of testing for a specific rToF patient, exploiting the advantages of each method, in order to obtain the maximal patient cooperation and, therefore, their accurate functional capacity. It is crucial to obtain the most robust measure of aerobic capacity in rToF patients during follow-up in order to risk-stratify for adverse events and to decide the optimal timing for PVR. Further studies on a wider cohort of patients are needed to confirm our findings, given the importance of establishing the most appropriate CPET modality for rToF patients.

## Figures and Tables

**Table 1:** All characteristics examined in 57 patients with rToF.

Demographic Features	Patient Characteristics	Mean (SD)	*n* (%)
	Male sex, *n* (%)		30 (53)
	Age	20.5 (7.8)	
	BMI	23.0 (3.3)	
	Age at surgery (months)	10.7 (10.7)	
	Type of surgery:		
	- Transannular patch	48 (84%)	
	- Infundibular patch	7(12%)	
	- Others (1 conduit, 1 valvulotomy)	2(4%)	
**MRI**	Age at MRI (years)	20.9 (7.3)	
	RVEDVi (ml/m^2^)	119.4 (22.2)	
	RVESVi (mL/m^2^)	53.4 (14.4)	
	RVEF (%)	54.9 (6.0)	
	LVEDVi (mL/m^2^)	80.9 (11.7)	
	LVESVi (mL/m^2^)	35.4 (7.9)	
	LVEF (%)	56.2 (5.3)	
	PR (%)	26.7 (17.3)	

Legend: BMI: body mass index; LVEDVi: left ventricular end-diastolic volume indexed to body surface area; LVEF: left ventricular ejection fraction; LVESVi: left ventricular end-systolic volume indexed to body surface area; PR: pulmonary regurgitation; RVEDVi: right ventricular end-diastolic volume indexed to body surface area; RVEF: right ventricular ejection fraction; RVESVi: right ventricular end-systolic volume indexed to body surface area.

**Table 2:** Comparison of the two methods of performing the CPET in 57 rToF patients. Legend: AT: anaerobic threshold; OUES: oxygen uptake efficiency slope; RER: respiratory exchange ratio; RCP: respiratory compensation point; VE/VCO_2_: ventilatory equivalent for CO_2_; VE: ventilation; VO_2_: oxygen consumption.

	Treadmill	Cycle Ergometer	*p*-Value
	Mean ± SD/Median (IQR)	Mean ± SD/Median (IQR)	
**Total duration (s) (s)**	607.0 (198.3)	570.0 (181.5)	***p* = 0.003**
**Peak RER**	1.10 (0.20)	1.10 (0.10)	*p* = 0.028
**Peak VO_2_ (mL/min)**	2054 ± 546	1684 ± 450	***p* < 0.001**
**VO_2_ max (mL/min/kg)**	31.7 (6.9)	25.5 (5.5)	***p* < 0.001**
**VO_2_ pred Wasserman (%)**	87.0 ± 15.0	70.0 ± 12.0	***p* < 0.001**
**VO_2_ pred Burstein (%)**	75.0 ± 22.0	64.0 ± 13.0	***p* < 0.001**
**VO_2_ at AT (mL/min/kg)**	22.0 ± 4.5	15.3 ± 3.9	***p* < 0.001**
**VE/VCO_2_ max**	30.4 (5.4)	32.2 (4.5)	***p* < 0.001**
**VE/VCO_2_ slope at RCP**	27.9 (3.7)	28.5 (4.6)	*p* = 0.150
**Peak O_2_ pulse (mL/beat)**	12.1 ± 3.4	10.6 ± 3.0	***p* < 0.001**
**OUES (mL/min/L/min)**	2292.0 (639.4)	1933.2 (623.6)	***p* < 0.001**

## Data Availability

The datasets that underpin the findings presented in this article are the property of the Bambino Gesù Children’s Hospital, IRCCS, due to privacy restriction.
